# Triglyceride glucose index is a significant predictor of severe disturbance of consciousness and all-cause mortality in critical cerebrovascular disease patients

**DOI:** 10.1186/s12933-023-01893-6

**Published:** 2023-06-29

**Authors:** Ting Chen, Yuan Qian, Xingli Deng

**Affiliations:** 1 Department of Neurosurgery, The First Affiliated Hospital of Kunming Medical University, Kunming, Yunnan, 650032 China; 2 Clinical Medical Research Center for Obstetrics and Gynecology (Yunnan Joint Key Laboratory), Kunming city of Maternal and Child Health Hospital,Kunming city of Women and Children Hospital, Kunming, Yunnan 650032 China; 3 Puer People’s Hospital, Puer, Yunnan 665099, China; 4 Yunnan Provincial Clinical Research Center for Neurological Disease, Kunming, Yunnan, 650032 China

**Keywords:** TyG index, Cerebral infarction, Non-traumatic cerebral hemorrhage, ICU, MIMIC database

## Abstract

**Objective:**

The association of the triglyceride-glucose (TyG) index with severe consciousness disturbance and in-hospital mortality in patients with cerebrovascular disease in the intensive care unit (ICU) is unclear. This study aimed to investigate the TyG index’s predictive ability on the severity of impaired consciousness and in-hospital mortality in patients with cerebrovascular disease in the ICU.

**Method:**

Patients diagnosed with non-traumatic cerebral hemorrhage and cerebral infarction were extracted from the MIMIC-IV database and analyzed as two cohorts. The association between the TyG index and the severity of patients’ impairment of consciousness and in-hospital mortality was analyzed using logistic regression models. Using restricted cubic spline curves, we analyzed potential nonlinear relationships between TyG indices and outcome indicators. receiver operating characteristic (ROC) curves were utilized to evaluate the predictive ability of the TyG index for outcome indicators.

**Result:**

The study’s last two cohorts comprised 537 patients with traumatic cerebral hemorrhage and 872 patients with cerebral infarction. TyG index was a significant predictor of the severity of impaired consciousness and in-hospital mortality in patients with cerebrovascular disease, as determined by logistic regression. The risk of severe consciousness impairment and in-hospital mortality increased roughly linearly with increasing TyG index.

**Conclusion:**

The TyG index was found to be a significant predictor for severe impairment of consciousness and in-hospital death in patients with cerebrovascular disease in the ICU, and it provides some predictive value for the severity of consciousness disturbances and in-hospital mortality in cerebrovascular disease patients.

## Background

Cerebrovascular disease remains a significant cause of the global disease burden [1]. Most patients who survive a cerebrovascular event suffer from disabilities and nerve damage and are at high risk for recurrent cerebrovascular events, and declining cognitive, and systemic vascular disease [2, 3]. Cerebrovascular disease patients admitted to the intensive care unit (ICU) frequently have more severe disorders of consciousness, a more complicated condition, and a higher mortality rate [4].

Insulin resistance (IR), defined as a decrease in the efficiency of insulin to promote glucose uptake and utilization, is a prominent feature of the metabolic syndrome [5] and has an impact on multiple organs’ metabolic signaling pathways [6]. Triglyceride-glucose (TyG) index has become a substitute marker for IR. Several studies have revealed the relevance of the TyG index and cardiovascular disease. A higher TyG index is linked to an increased risk of cardiovascular events, cerebrovascular events, and kidney microvascular damage [7–9], as well as inflammation, endothelial dysfunction, oxidative stress, and prothrombotic state [10, 11]. There are also studies demonstrating the prognostic value of the TyG index in heart failure and severe illnesses [12]. There are currently no studies that directly illustrate the connection between IR and disturbance consciousness. Nonetheless, several pathophysiological processes may explain the potential effects of IR on brain. Metabolic disturbances can result in hyperviscosity and an increase in the brain’s water content, leading to a decrease in cerebral blood flow and intracellular acidosis [13–15]. It has also been demonstrated that increased nitrogen waste in the brains of hyperglycemic patients may cause toxic and metabolic damage to brain tissue, particularly the basal ganglia, affecting focal cellular metabolism and leading to cellular edema while increasing the permeability of the blood-brain barrier. The severity of the symptoms ranges from mild (drowsiness) to severe (coma)[16, 17]. Taken together, we can hypothesize that IR exacerbates the disturbance of consciousness or increases the mortality rate of critical patients with cerebrovascular disease. As an alternative index of IR, the TyG index may be helpful for predicting patients’ consciousness disturbance and mortality. However, the validity of this hypothesis is unclear.

Therefore, this study aimed to examine whether the TyG index can be used as a predictor of severe disturbance of consciousness and in-hospital mortality in patients with non-traumatic cerebral hemorrhage and cerebral infarction in the ICU. This may help to distinguish patients at higher risk for more close monitoring or early intervention.

## Method

### Study population

The original data were derived from the MIMIC-IV database. The MIMIC-IV database is a comprehensive single-center database maintained by the Massachusetts Institute of Technology Lab for Computational Physiology [18]. The author (Ting Chen) was granted access to the dataset (ID: 10,946,391) and is responsible for data extraction.

Inclusion criteria: a diagnosis of non-traumatic cerebral hemorrhage or cerebral infarction (based on the International Classification of Diseases, Ninth Revision (ICD-9), or International Classification of Diseases, Tenth Revision (ICD-10)). Exclusion criteria: (a) not admitted to an ICU, (b) missing triglyceride and glucose data, (c) severe disorders of consciousness prior to hospital admission (Glasgow coma scale score < 8), (d) follow-up time less than 1 day. For patients with multiple admissions, we chose the last admission.

### Patient characteristics

Structured query language (SQL) was used to extract demographic information, laboratory indicators, comorbidities, intra-hospital mortality, and scores from the MIMIC-IV database. Demographic information included gender, age, race, and body mass index (BMI). Laboratory indicators included triglyceride, glucose, creatine phosphokinase (CPK), creatine kinase isoenzyme MB (CKMB), lactate dehydrogenase (IDH), alkaline phosphatase (ALP), aspartate aminotransferase (AST), international normalized ratio (INR), alanine aminotransferase (ALT), creatinine, prothrombin Time (PT), active partial thromboplastin time (PTT), glycated hemoglobin A 1c (HbA 1c). Comorbidities and personal history of patients were identified based on ICD-9 and ICD-10, including anemia, cancer, chronic kidney disease (CKD), hyperlipidemia, diabetes, hypertension, respiratory failure, long-term use of antiplatelet agents/anticoagulants, alcohol abuse, and tobacco use. Scores include Glasgow coma scale (GCS) score, acute physiology score III (APSIII), oxford acute severity of illness score (OASIS) and simplified acute physiology score (SAPSII). TyG index was calculated according to the formula: ln [fasting triglycerides (mg/dL) × fasting glucose (mg/dL)]/2. For laboratory indicators that were measured multiple times within 24 h of admission, the first measurement was used in this study. To reduce reverse causation bias, data that were collected after the outcome events were deemed invalid. Missing values for laboratory indicators are common in the MIMIC-IV database. To reduce the bias caused by sample exclusion, we calculated the percentage of missing values for each continuous variable. For variables with a missing value share of less than 20%, we predicted the missing values using a random forest-based multiple imputation method, predicting five outcomes, and calculating the mean of the five outcomes as the final result. For variables with missing value proportion greater than 20%, we classified the missing values. For non-normally distributed continuous variables, we analyzed them after converting them into categorical variables according to the normal reference range provided by the MIMIC-IV database.

### Outcome measures

The primary outcome indicator was the occurrence of severe impairment of consciousness, defined as a GCS score of less than 8, within 30 days of patient admission. the secondary outcome indicator was the patient’s in-hospital mortality within 30 days.

### Statistical analysis

Continuous variables are presented as the mean (standard deviation (SD)) or median (interquartile range, (IQR)), and were compared using a student t-test or a nonparametric test, as appropriate. Categorical variables are presented as frequencies and percentages (%) and were compared between groups using the Pearson chi-square test or Fisher’s exact test. Logistic regression models were used to estimate odds ratios (ORs) and their 95% confidence intervals (95% CIs) and adjusted for several confounding variables (model 1: unadjusted; Model 2: adjusted for age, sex, BMI, and race; model 3: adjusted for age, sex, BMI, race, CPK, CKMB, IDH, ALP, AST, INR, ALT, creatinine, PT, PTT, HbA 1c, anemia, cancer, CKD, hyperlipemia, hypertension, respiratory failure, diabetes, alcohol abuse, long-term use of antiplatelet agents/anticoagulants, tobacco use.). To examine the incidence of outcome events according to various levels of the TyG index, we included TyG index segments as categorical variables according to quartiles in the model (the lowest quartile of TyG index values was used as the reference group). To prevent multicollinearity, variables having a variance inflation factor of more than 5 were excluded from the model. Potential nonlinear correlations between TyG index levels and outcomes were investigated utilizing restricted cubic splines. The area under the ROC curve (AUC) was calculated to evaluate the predictive ability of the numerical- and categorical-type TyG index individually. Clinical decision curves were plotted, and the integrated discrimination improvement (IDI) were calculated separately in order to assess the improvement in the predictive ability and clinical value of scoring tools resulting from the incorporation of TyG indices. Two-tailed P values < 0.05 were considered statistically significant, and all statistical analyses were performed using the R language (R version 4.2.2).

## Result

### Study population characteristics

**Fig. 1:**
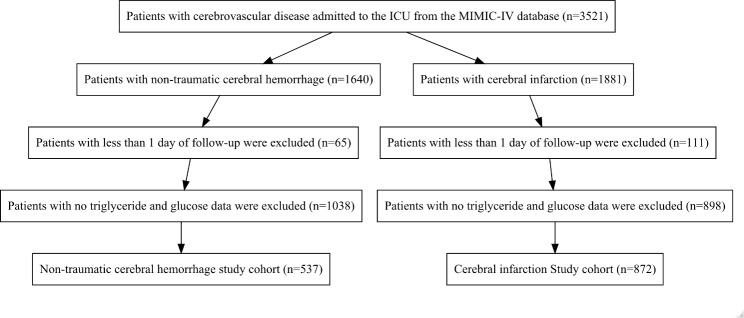
Flowchart of patient selection

A total of 1409 patients were enrolled in the present study (537 patients with non-traumatic cerebral hemorrhage and 872 patients with cerebral infarction). The patient selection process is outlined in Fig. 1. The median age of patients in both groups was 71 years. There were 293 (54.6%) men and 244 (45.4%) women with non-traumatic cerebral hemorrhage, of which 48 (8.9%) developed severe disturbance of consciousness and 99 (18.4%) died in the hospital (Table 1). There were 430 (49.3%) males and 442 (50.7%) females with cerebral infarction, a total of 161 (18.5%) with severe disturbance of consciousness, and 129 (14.8%) in-hospital deaths (Table 2). Patients with non-traumatic cerebral hemorrhage and patients with cerebral infarction were described based on the admission TyG index quartiles (Tables 1 and 2).

**Table 1:** All characteristics of patients with nontraumatic cerebral hemorrhage

Characteristics	Overall	Q1	Q2	Q3	Q4	P
n	537	135	135	134	133	
In-hospital death = Yes (%)	99 (18.4)	12 (8.9)	24 (17.8)	18 (13.4)	45 (33.8)	**< 0.001**
Hospital stays (day)	8.00 [4.00, 16.00]	7.00 [4.00, 12.00]	8.00 [4.00, 13.50]	8.00 [5.00, 17.00]	10.00 [4.00, 22.00]	**0.039**
GCS score < 8 (%)	48 (8.9)	5 (3.7)	13 (9.6)	11 (8.2)	19 (14.3)	**0.025**
Follow-up time of GCS score	7.00 [4.00, 14.00]	7.00 [4.00, 11.50]	6.00 [3.00, 12.00]	7.00 [4.00, 15.00]	8.00 [4.00, 19.00]	0.131
Gender = Male (%)	293 (54.6)	67 (49.6)	77 (57.0)	75 (56.0)	74 (55.6)	0.609
Race (%)						0.215
Asian	14 (2.6)	2 (1.5)	3 (2.2)	5 (3.7)	4 (3.0)	
Black	51 (9.5)	15 (11.1)	16 (11.9)	10 (7.5)	10 (7.5)	
Other	165 (30.7)	31 (23.0)	37 (27.4)	47 (35.1)	50 (37.6)	
White	307 (57.2)	87 (64.4)	79 (58.5)	72 (53.7)	69 (51.9)	
Anemia = Yes (%)	103 (19.2)	18 (13.3)	24 (17.8)	30 (22.4)	31 (23.3)	0.139
Cancer = Yes (%)	42 (7.8)	11 (8.1)	9 (6.7)	10 (7.5)	12 (9.0)	0.906
CKD = Yes (%)	92 (17.1)	17 (12.6)	23 (17.0)	26 (19.4)	26 (19.5)	0.393
Diabetes = Yes (%)	154 (28.7)	17 (12.6)	36 (26.7)	39 (29.1)	62 (46.6)	**< 0.001**
Hyperlipemia = Yes (%)	37 (6.9)	8 (5.9)	9 (6.7)	11 (8.2)	9 (6.8)	0.903
Hypertension = Yes (%)	449 (83.6)	107 (79.3)	121 (89.6)	117 (87.3)	104 (78.2)	**0.022**
Respiratory failure = Yes (%)	51 (9.5)	9 (6.7)	6 (4.4)	15 (11.2)	21 (15.8)	**0.008**
Site (%)						**0.029**
cerebellum	30 (5.6)	9 (6.7)	8 (5.9)	5 (3.7)	8 (6.0)	
cortical	121 (22.5)	35 (25.9)	34 (25.2)	27 (20.1)	25 (18.8)	
intraventricular	79 (14.7)	17 (12.6)	15 (11.1)	23 (17.2)	24 (18.0)	
Other	216 (40.2)	57 (42.2)	62 (45.9)	54 (40.3)	43 (32.3)	
subarachnoid	61 (11.4)	6 (4.4)	11 (8.1)	19 (14.2)	25 (18.8)	
subdural	30 (5.6)	11 (8.1)	5 (3.7)	6 (4.5)	8 (6.0)	
Alcohol abuse = Yes (%)	56 (10.4)	10 (7.4)	6 (4.4)	23 (17.2)	17 (12.8)	**0.003**
Long-term use of antiplatelet agents/anticoagulants = Yes (%)	123 (22.9)	30 (22.2)	36 (26.7)	30 (22.4)	27 (20.3)	0.648
Tobacco use = Yes (%)	6 (1.1)	0 (0.0)	1 (0.7)	3 (2.2)	2 (1.5)	0.333
BMI (%)						
< 18	6 (1.1)	1 (0.7)	1 (0.7)	2 (1.5)	2 (1.5)	0.874
> 24	143 (26.6)	41 (30.4)	38 (28.1)	26 (19.4)	38 (28.6)	0.174
18–24	44 (8.2)	17 (12.6)	11 (8.1)	10 (7.5)	6 (4.5)	0.113
CPK (%)						
≤ 130 IU/L	161 (30.0)	44 (32.6)	40 (29.6)	44 (32.8)	33 (24.8)	0.447
> 130 IU/L	171 (31.8)	39 (28.9)	44 (32.6)	35 (26.1)	53 (39.8)	0.089
CKMB (%)						
> 5 ng/mL	78 (14.5)	11 (8.1)	17 (12.6)	20 (14.9)	30 (22.6)	**0.008**
≤ 5 ng/mL	223 (41.5)	61 (45.2)	60 (44.4)	53 (39.6)	49 (36.8)	0.451
IDH (%)						
≤ 300 IU/L	244 (45.4)	71 (52.6)	63 (46.7)	56 (41.8)	54 (40.6)	0.184
> 300 IU/L	98 (18.2)	15 (11.1)	19 (14.1)	26 (19.4)	38 (28.6)	**0.001**
CRP (%)						
≤ 5 mg/L	49 (9.1)	18 (13.3)	13 (9.6)	10 (7.5)	8 (6.0)	0.178
> 10 mg/L	69 (12.8)	16 (11.9)	14 (10.4)	17 (12.7)	22 (16.5)	0.478
5–10 mg/L	22 (4.1)	3 (2.2)	10 (7.4)	4 (3.0)	5 (3.8)	0.143
HbA 1c (%)						**< 0.001**
4–6%	334 (62.2)	112 (83.0)	92 (68.1)	79 (59.0)	51 (38.3)	
> 6%	203 (37.8)	23 (17.0)	43 (31.9)	55 (41.0)	82 (61.7)	
Age (%)						**< 0.001**
< 40 years	20 (3.7)	5 (3.7)	3 (2.2)	5 (3.7)	7 (5.3)	
≥ 80 years	131 (24.4)	49 (36.3)	36 (26.7)	26 (19.4)	20 (15.0)	
40–59 years	100 (18.6)	20 (14.8)	12 (8.9)	31 (23.1)	37 (27.8)	
60–79 years	286 (53.3)	61 (45.2)	84 (62.2)	72 (53.7)	69 (51.9)	
ALP (%)						**0.003**
< 40 IU/L	13 (2.4)	4 (3.0)	5 (3.7)	0 (0.0)	4 (3.0)	
> 130 IU/L	65 (12.1)	7 (5.2)	14 (10.4)	17 (12.7)	27 (20.3)	
40–130 IU/L	459 (85.5)	124 (91.9)	116 (85.9)	117 (87.3)	102 (76.7)	
AST (%)						**0.003**
≤ 40 IU/L	384 (71.5)	110 (81.5)	101 (74.8)	90 (67.2)	83 (62.4)	
>40 IU/L	153 (28.5)	25 (18.5)	34 (25.2)	44 (32.8)	50 (37.6)	
Creatinine (%)						**0.007**
< 0.5 mg/dL	9 (1.7)	3 (2.2)	1 (0.7)	2 (1.5)	3 (2.3)	
> 1.2 mg/dL	108 (20.1)	17 (12.6)	21 (15.6)	29 (21.6)	41 (30.8)	
0.5–1.2 mg/dL	420 (78.2)	115 (85.2)	113 (83.7)	103 (76.9)	89 (66.9)	
INR (%)						0.859
0.9–1.1	285 (53.1)	75 (55.6)	71 (52.6)	72 (53.7)	67 (50.4)	
> 1.1	252 (46.9)	60 (44.4)	64 (47.4)	62 (46.3)	66 (49.6)	
PT (%)						0.094
< 11 s	49 (9.1)	11 (8.1)	7 (5.2)	21 (15.7)	10 (7.5)	
> 13 s	183 (34.1)	50 (37.0)	46 (34.1)	40 (29.9)	47 (35.3)	
11–13 s	305 (56.8)	74 (54.8)	82 (60.7)	73 (54.5)	76 (57.1)	
PTT (%)						0.263
< 25 s	81 (15.1)	17 (12.6)	17 (12.6)	20 (14.9)	27 (20.3)	
> 37 s	35 (6.5)	13 (9.6)	6 (4.4)	7 (5.2)	9 (6.8)	
25–37 s	421 (78.4)	105 (77.8)	112 (83.0)	107 (79.9)	97 (72.9)	
ALT (%)						**< 0.001**
≤ 40 IU/L (%)	444 (82.7)	122 (90.4)	119 (88.1)	108 (80.6)	95 (71.4)	
>40 IU/L (%)	93 (17.3)	13 (9.6)	16 (11.9)	26 (19.4)	38 (28.6)	

TyG index: Q1 (7.129–8.378), Q2 (8.378–8.764), Q3 (8.764–9.239), Q4 (9.239–12.102)

**Table 2:** All characteristics of patients with cerebral infarction

	Overall	Q1	Q2	Q3	Q4	P
n	872	218	218	218	218	
In-hospital death = Yes (%)	129 (14.8)	22 (10.1)	24 (11.0)	32 (14.7)	51 (23.4)	**< 0.001**
Hospital stays (day)	7.00 [4.00, 13.00]	6.00 [3.00, 10.00]	7.00 [4.00, 12.00]	7.00 [4.00, 14.00]	9.00 [4.00, 19.75]	**0.001**
GCS score < 8 (%)	161 (18.5)	28 (12.8)	40 (18.3)	38 (17.4)	55 (25.2)	**0.010**
Follow-up time of GCS score	5.00 [3.00, 10.00]	5.00 [3.00, 9.00]	5.00 [3.00, 10.00]	5.00 [3.00, 10.00]	6.00 [3.00, 13.00]	0.216
Gender = Male (%)	430 (49.3)	104 (47.7)	110 (50.5)	110 (50.5)	106 (48.6)	0.92
Race (%)						0.554
Asian	23 (2.6)	4 (1.8)	5 (2.3)	9 (4.1)	5 (2.3)	
Black	79 (9.1)	25 (11.5)	17 (7.8)	17 (7.8)	20 (9.2)	
Other	273 (31.3)	60 (27.5)	67 (30.7)	69 (31.7)	77 (35.3)	
White	497 (57.0)	129 (59.2)	129 (59.2)	123 (56.4)	116 (53.2)	
Anemia = Yes (%)	208 (23.9)	40 (18.3)	52 (23.9)	58 (26.6)	58 (26.6)	0.141
Cancer = Yes (%)	80 (9.2)	17 (7.8)	24 (11.0)	13 (6.0)	26 (11.9)	0.109
CKD = Yes (%)	156 (17.9)	39 (17.9)	38 (17.4)	30 (13.8)	49 (22.5)	0.128
Diabetes = Yes (%)	303 (34.7)	28 (12.8)	58 (26.6)	79 (36.2)	138 (63.3)	**< 0.001**
Hyperlipemia = Yes (%)	79 (9.1)	16 (7.3)	23 (10.6)	25 (11.5)	15 (6.9)	0.245
Hypertension = Yes (%)	693 (79.5)	162 (74.3)	173 (79.4)	177 (81.2)	181 (83.0)	0.13
Respiratory failure = Yes (%)	88 (10.1)	16 (7.3)	22 (10.1)	20 (9.2)	30 (13.8)	0.154
Site (%)						**0.023**
Basilar artery	15 (1.7)	1 (0.5)	8 (3.7)	5 (2.3)	1 (0.5)	
Bilateral cerebellar artery	11 (1.3)	2 (0.9)	3 (1.4)	3 (1.4)	3 (1.4)	
Left anterior cerebral artery	13 (1.5)	5 (2.3)	3 (1.4)	1 (0.5)	4 (1.8)	
Left cerebellar artery	15 (1.7)	3 (1.4)	6 (2.8)	4 (1.8)	2 (0.9)	
Left carotid artery	27 (3.1)	6 (2.8)	5 (2.3)	9 (4.1)	7 (3.2)	
Left middle cerebral artery	160 (18.3)	54 (24.8)	39 (17.9)	37 (17.0)	30 (13.8)	
Left posterior cerebral artery	23 (2.6)	4 (1.8)	8 (3.7)	3 (1.4)	8 (3.7)	
Other	399 (45.8)	83 (38.1)	93 (42.7)	98 (45.0)	125 (57.3)	
Right anterior cerebral artery	4 (0.5)	0 (0.0)	2 (0.9)	2 (0.9)	0 (0.0)	
Right cerebellar artery	16 (1.8)	3 (1.4)	3 (1.4)	5 (2.3)	5 (2.3)	
Right carotid artery	31 (3.6)	9 (4.1)	7 (3.2)	6 (2.8)	9 (4.1)	
Right middle cerebral artery	143 (16.4)	46 (21.1)	35 (16.1)	41 (18.8)	21 (9.6)	
Right posterior cerebral artery	15 (1.7)	2 (0.9)	6 (2.8)	4 (1.8)	3 (1.4)	
Alcohol abuse = Yes (%)	49 (5.6)	9 (4.1)	12 (5.5)	13 (6.0)	15 (6.9)	0.654
Long-term use of antiplatelet agents/anticoagulants = Yes (%)	270 (31.0)	74 (33.9)	71 (32.6)	72 (33.0)	53 (24.3)	0.106
Tobacco use = Yes (%)	18 (2.1)	8 (3.7)	4 (1.8)	2 (0.9)	4 (1.8)	0.23
BMI (%)						
< 18	7 (0.8)	2 (0.9)	3 (1.4)	2 (0.9)	0 (0.0)	0.434
> 24	314 (36.0)	80 (36.7)	73 (33.5)	69 (31.7)	92 (42.2)	0.108
18–24	88 (10.1)	29 (13.3)	28 (12.8)	15 (6.9)	16 (7.3)	**0.035**
CPK (%)						
≤ 130 IU/L	287 (32.9)	65 (29.8)	76 (34.9)	72 (33.0)	74 (33.9)	0.699
> 130 IU/L	211 (24.2)	55 (25.2)	44 (20.2)	57 (26.1)	55 (25.2)	0.454
CKMB (%)						
> 5 ng/mL	110 (12.6)	27 (12.4)	23 (10.6)	26 (11.9)	34 (15.6)	0.439
≤ 5 ng/mL	337 (38.6)	89 (40.8)	90 (41.3)	77 (35.3)	81 (37.2)	0.513
IDH (%)						
≤ 300 IU/L	379 (43.5)	106 (48.6)	102 (46.8)	86 (39.4)	85 (39.0)	0.088
> 300 IU/L	164 (18.8)	21 (9.6)	32 (14.7)	49 (22.5)	62 (28.4)	**< 0.001**
CRP (%)						
≤ 5 mg/L	78 (8.9)	33 (15.1)	18 (8.3)	19 (8.7)	8 (3.7)	**< 0.001**
> 10 mg/L	103 (11.8)	23 (10.6)	31 (14.2)	18 (8.3)	31 (14.2)	0.144
5–10 mg/L	35 (4.0)	8 (3.7)	6 (2.8)	11 (5.0)	10 (4.6)	0.625
HbA 1c (%)						**< 0.001**
4–6%	547 (62.7)	187 (85.8)	158 (72.5)	131 (60.1)	71 (32.6)	
> 6%	325 (37.3)	31 (14.2)	60 (27.5)	87 (39.9)	147 (67.4)	
Age (%)						**< 0.001**
< 40 years	32 (3.7)	10 (4.6)	6 (2.8)	9 (4.1)	7 (3.2)	
≥80 years	233 (26.7)	74 (33.9)	70 (32.1)	59 (27.1)	30 (13.8)	
40–59 years	174 (20.0)	31 (14.2)	36 (16.5)	45 (20.6)	62 (28.4)	
60–79 years	433 (49.7)	103 (47.2)	106 (48.6)	105 (48.2)	119 (54.6)	
ALP (%)						0.667
< 40 IU/L	20 (2.3)	6 (2.8)	5 (2.3)	4 (1.8)	5 (2.3)	
> 130 IU/L	132 (15.1)	27 (12.4)	31 (14.2)	33 (15.1)	41 (18.8)	
40–130 IU/L	720 (82.6)	185 (84.9)	182 (83.5)	181 (83.0)	172 (78.9)	
AST (%)						0.352
≤ 40 IU/L (%)	601 (68.9)	160 (73.4)	150 (68.8)	143 (65.6)	148 (67.9)	
> 40 IU/L (%)	271 (31.1)	58 (26.6)	68 (31.2)	75 (34.4)	70 (32.1)	
Creatinine (%)						**0.026**
< 0.5 mg/dL	10 (1.1)	2 (0.9)	4 (1.8)	3 (1.4)	1 (0.5)	
> 1.2 mg/dL	203 (23.3)	43 (19.7)	45 (20.6)	45 (20.6)	70 (32.1)	
0.5–1.2 mg/dL	659 (75.6)	173 (79.4)	169 (77.5)	170 (78.0)	147 (67.4)	
INR (%)						0.795
0.9–1.1	436 (50.0)	109 (50.0)	111 (50.9)	113 (51.8)	103 (47.2)	
>1.1	436 (50.0)	109 (50.0)	107 (49.1)	105 (48.2)	115 (52.8)	
PT (%)						0.46
< 11 s	73 (8.4)	19 (8.7)	19 (8.7)	12 (5.5)	23 (10.6)	
> 13 s	352 (40.4)	84 (38.5)	93 (42.7)	95 (43.6)	80 (36.7)	
11–13 s	447 (51.3)	115 (52.8)	106 (48.6)	111 (50.9)	115 (52.8)	
PTT (%)						0.382
< 25 s	100 (11.5)	22 (10.1)	22 (10.1)	28 (12.8)	28 (12.8)	
> 37 s	125 (14.3)	24 (11.0)	36 (16.5)	28 (12.8)	37 (17.0)	
25–37 s	647 (74.2)	172 (78.9)	160 (73.4)	162 (74.3)	153 (70.2)	
ALT (%)						0.204
≤ 40 IU/L (%)	712 (81.7)	185 (84.9)	182 (83.5)	176 (80.7)	169 (77.5)	
> 40 IU/L (%)	160 (18.3)	33 (15.1)	36 (16.5)	42 (19.3)	49 (22.5)	

TyG index: Q1 (7.129–8.428), Q2 (8.428–8.822); Q3 (8.822–9.251); Q4 (9.251–12.102)

In-hospital mortality (P < 0.001), length of stay (P = 0.039), and incidence of severe disruption of consciousness (P = 0.025), according to data from patients with non-traumatic cerebral hemorrhage, increased as the TyG index rose. Patients with alcohol abuse had higher levels of TyG index. Patients with higher TyG index levels were more likely to have comorbid conditions like diabetes (P < 0.001), hypertension (P = 0.022), respiratory failure (P = 0.008), as well as higher levels of HbA 1c (P < 0.001), CKMB (P = 0.008), IDH (P = 0.001), ALP (P = 0.003), AST (P = 0.003), creatinine (P = 0.007), and ALT (P < 0.001) (Table 1).

Patients with cerebral infarction exhibited a gradual increase in in-hospital mortality (P < 0.001), length of stay (P = 0.001), and incidence of severe disorder of consciousness (P = 0.010) as the level of TyG index increased, as well as a gradual decrease in patients with a normal BMI (18–24 kg/m^2^) as the level of TyG index increased. Patients with a higher TyG index were younger (P < 0.001), more likely to combine diabetes (P < 0.001), and had higher levels of HbA 1c (P < 0.001), IDH (P < 0.001), and creatinine (P = 0.026) (Table 2).

### Influence of TyG index on the severity of consciousness disturbance and in-hospital mortality

The logistic regression model was used to assess the effect of exposure variables on the outcome measures, adjusting for covariates. (Model 1: unadjusted. Model 2: adjusted for age, sex, BMI, and race. Model 3: adjusted for age, sex, BMI, race, CPK, CKMB, IDH, ALP, AST, INR, ALT, creatinine, PT, PTT, anemia, cancer, CKD, hyperlipemia, hypertension, respiratory failure, diabetes.)

Analysis of patients with nontraumatic cerebral hemorrhage showed that in unadjusted (OR [95% CI], 4.33 [1.68, 13.4]. P-value = 0.005), adjusted by demographic information (OR [95% CI], 4.34 [1.63, 13.8], P-value = 0.006) and fully adjusted (OR [95% CI], 3.68 [1.12, 13.8], P-value = 0.040) models, higher TyG index (Q4: 9.239–12.102) patients had a significantly higher risk of severe disturbance of consciousness than those with a lower TyG index (Q1: 7.129–8.378). However, as the model was adjusted, the effect of the TyG index on the severity of consciousness disorder gradually decreased. In addition, the results demonstrated that a higher TyG index was significantly associated with patients’ in-hospital mortality. In unadjusted (Q2: OR [95% CI], 2.22 [1.08, 4.78], P-value = 0.035; Q4: OR [95% CI], 5.24 [2.70, 10.9], P-value < 0.001), adjusted by demographic information (Q2: OR [95% CI], 2.41 [1.15, 5.30]. P-value = 0.023; Q4: OR [95% CI], 5.65 [2.81, 12.2], P-value < 0.001) and fully adjusted (Q2: OR [95% CI], 3.81 [1.57, 9.84], P-value = 0.004; Q4: OR [95% CI], 8.86 [3.49, 24.2], P-value < 0.001) models, patients with a TyG index of Q2 and Q4 had a significantly higher risk of in-hospital death than Q1 (Table 3).

**Table 3:** Logistic regression analysis of consciousness disturbance and in-hospital mortality in cerebrovascular disease patients

Factor	OR^*1*^	95% CI^*1*^	P	OR^*1*^	95% CI^*1*^	P	OR^*1*^	95% CI^*1*^	P
	Model 1			Model 2			Model 3		
Severity of consciousness disturbance (patients with non-traumatic cerebral hemorrhage)									
TyG^*2*^	P for trend: 0.074			P for trend: 0.117			P for trend: 0.594		
Q1	—	—		—	—		—	—	
Q2	2.77	1.01, 8.84	0.060	2.69	0.97, 8.70	0.072	2.60	0.84, 9.21	0.110
Q3	2.33	0.82, 7.56	0.130	2.04	0.69, 6.83	0.200	1.52	0.44, 5.73	0.500
Q4	4.33	1.68, 13.4	**0.005**	4.34	1.63, 13.8	**0.006**	3.68	1.12, 13.8	**0.040**
Severity of consciousness disturbance (patients with cerebral infarction)									
TyG^*3*^	P for trend: 0.014			P for trend: 0.002			P for trend: 0.168		
Q1	—	—		—	—		—	—	
Q2	1.52	0.91, 2.60	0.110	1.54	0.91, 2.65	0.110	1.47	0.82, 2.67	0.200
Q3	1.43	0.85, 2.45	0.200	1.48	0.87, 2.56	0.200	1.15	0.62, 2.14	0.700
Q4	2.29	1.40, 3.82	**0.001**	2.78	1.66, 4.74	**< 0.001**	2.48	1.31, 4.74	**0.005**
In-hospital mortality (patients with non-traumatic cerebral hemorrhage)									
TyG^*2*^	P for trend: <0.001			P for trend: <0.001			P for trend: <0.001		
Q1	—	—		—	—		—	—	
Q2	2.22	1.08, 4.78	**0.035**	2.41	1.15, 5.30	**0.023**	3.81	1.57, 9.84	**0.004**
Q3	1.59	0.74, 3.53	0.200	1.72	0.78, 3.91	0.200	2.32	0.90, 6.23	0.085
Q4	5.24	2.70, 10.9	**< 0.001**	5.65	2.81, 12.2	**< 0.001**	8.86	3.49, 24.2	**< 0.001**
In-hospital mortality (patients with cerebral infarction)									
TyG^*3*^	P for trend: <0.001			P for trend: <0.001			P for trend: 0.016		
Q1	—	—		—	—		—	—	
Q2	1.1	0.60, 2.04	0.800	1.11	0.59, 2.07	0.700	0.87	0.44, 1.73	0.700
Q3	1.53	0.86, 2.76	0.150	1.63	0.91, 2.98	0.100	1.31	0.67, 2.61	0.400
Q4	2.72	1.60, 4.75	**< 0.001**	3.06	1.77, 5.47	**< 0.001**	2.51	1.26, 5.10	**0.010**

^*1*^ OR = Odds Ratio, CI = Confidence Interval

^*2*^ TyG index: Q1 (7.129–8.378), Q2 (8.378–8.764), Q3 (8.764–9.239), Q4 (9.239–12.102)

^*3*^ TyG index: Q1 (7.129–8.428), Q2 (8.428–8.822); Q3 (8.822–9.251); Q4 (9.251–12.102)

Model 1: unadjusted

Model 2: adjusted for age, sex, BMI, and race.

Model 3: adjusted for age, sex, BMI, race, CPK, CKMB, IDH, ALP, AST, INR, ALT, Creatinine, PT, PTT, HbA1c, anemia, cancer, CKD, hyperlipemia, hypertension, respiratory failure, diabetes, alcohol abuse, long-term use of antiplatelet agents/anticoagulants, tobacco use.

Analysis of patients with cerebral infarction showed that high levels of TyG index (Q4: 9.251–12.102) were significantly associated with the severity of consciousness disturbance in unadjusted (OR [95% CI], 2.29 [1.40, 3.82], P-value = 0.001), adjusted by demographic information (OR [95% CI], 2.78 [1.66, 4.74], P-value < 0.001) and fully adjusted (OR [95% CI], 2.48 [1.31, 4.74], P-value = 0.005) models. In addition, in unadjusted (OR [95% CI], 2.72 [1.60, 4.75], P-value < 0.001), adjusted by demographic information (OR [95% CI], 3.06 [1.77, 5.47], P-value < 0.001) and fully adjusted (OR [95% CI], 2.51 [1.26, 5.10], P-value = 0.010) models, patients with high TyG index (Q4: 9.251–12.102) had a significantly higher risk of in-hospital death than patients with low TyG index (Q2: 7.129–8.428) (Table 3).

**Fig. 2:**
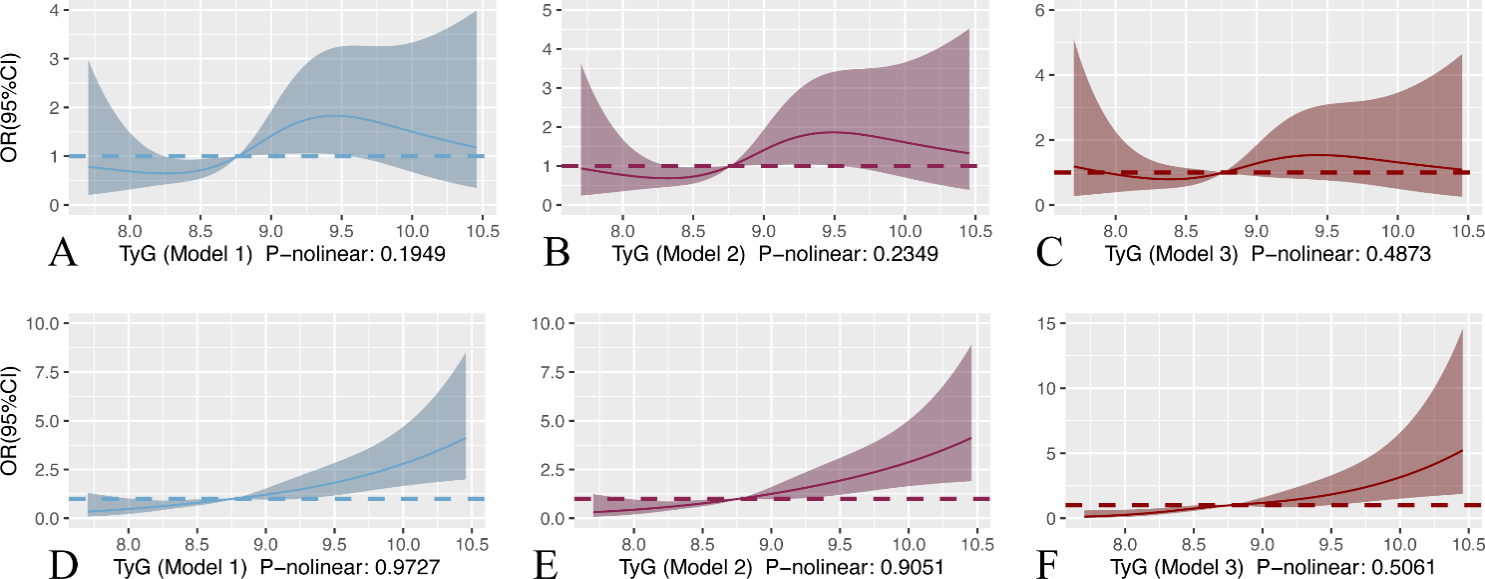
RCS curve of TyG index and OR in patients with nontraumatic cerebral hemorrhage: (**A**, **B**, and **C**) RCS curve for severe disturbance of consciousness. (**D**, **E**, and **F**) Restricted cubic spline for hospital mortality

**Fig. 3:**
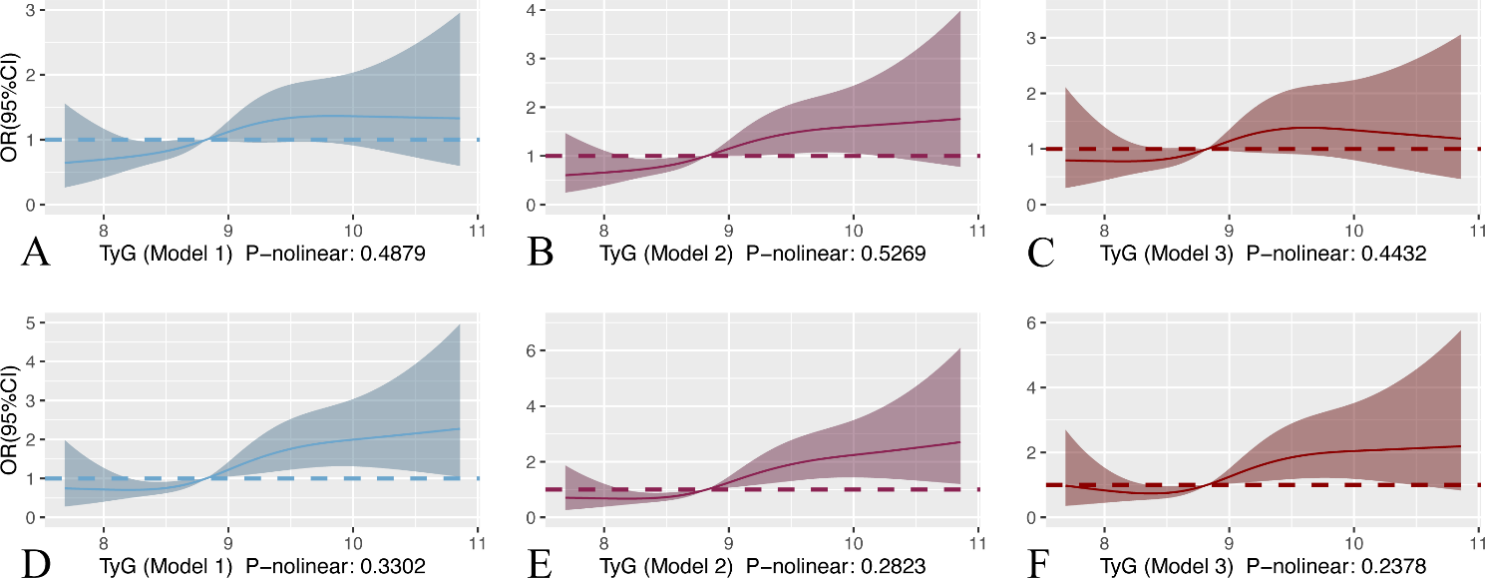
RCS curve of TyG index and OR in patients with cerebral infarction: (**A**, **B**, and **C**) RCS curve for severe disturbance of consciousness. (**D**, **E**, and **F**) RCS curve for hospital mortality

Alternately, we used restricted cubic splines (RCS) to assess possible nonlinearity in the association of the TyG index with outcomes. The result of RCS showed that the P values for nonlinearity for the TyG index outcomes were nonsignificant (P-nonlinear > 0.05). In patients with non-traumatic cerebral hemorrhage, the risk of severe consciousness disturbance increased approximately linearly with increasing TyG index levels when they were in the range of 8.75–9.40 (model 1: P-nonlinear 0.1949; model 2: P-nonlinear 0.2349; model 3: P-nonlinear 0.4873). When the TyG index was higher than 8.75, the risk of patient mortality in the hospital increased approximately linearly (model 1: P-nonlinear 0.9727; model 2: P-nonlinear 0.9051; model 3: P-nonlinear 0.5061) with increasing TyG index (Fig. 2). In patients with cerebral infarction, the risk of severe disturbance of consciousness (model 1: P-nonlinear 0.4879; model 2: P-nonlinear 0.5269; model 3: P-nonlinear 0.4432) and in-hospital death (model 1: P-nonlinear 0.3302; model 2: P-nonlinear 0.2823; model 3: P-nonlinear 0.2378) increased approximately linearly with TyG index levels (Fig. 3).

### The predictive ability and incremental effect of the TyG index

**Fig. 4:**
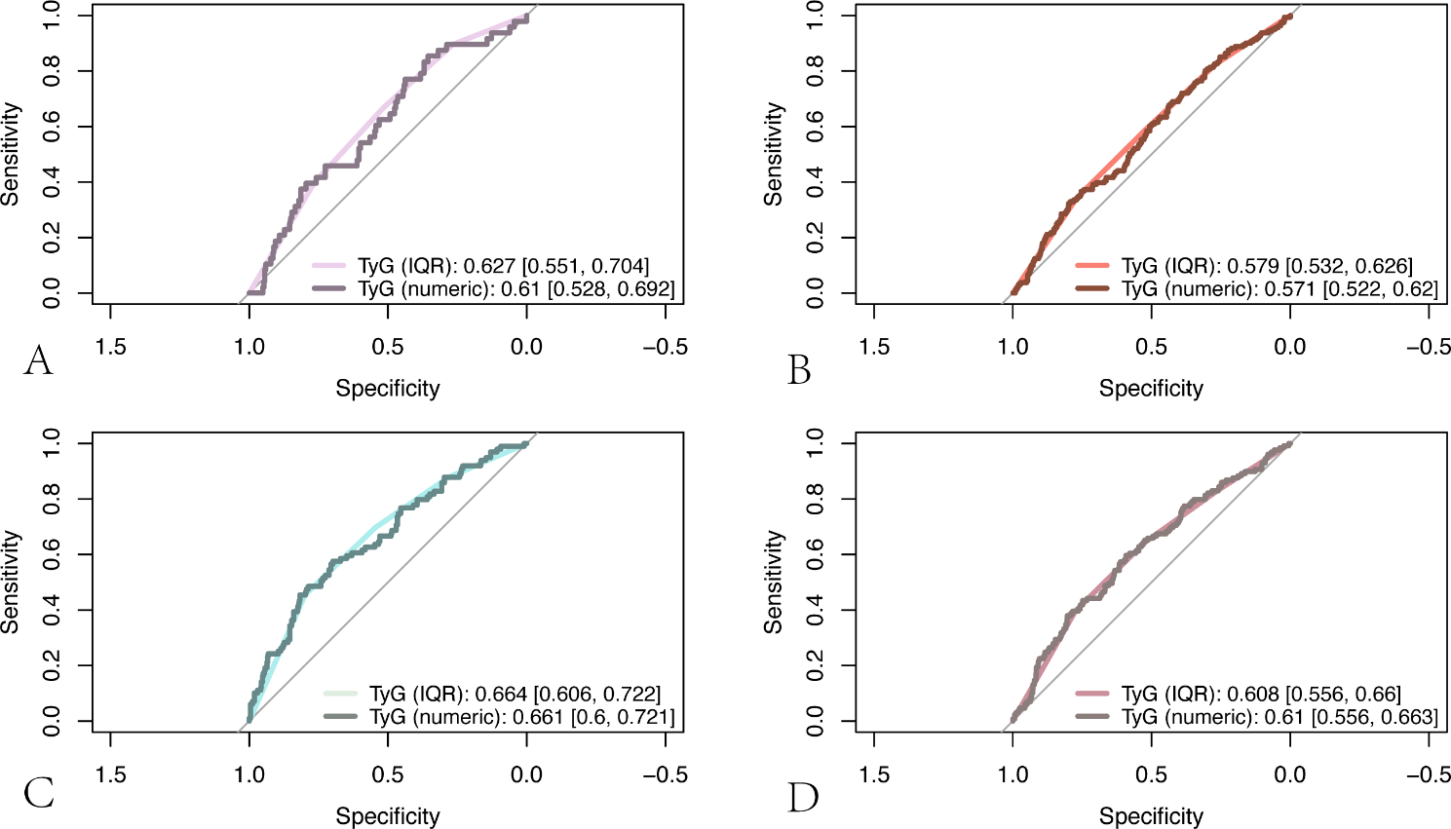
(**A** and **B**) ROC curve analysis of TyG index predicting severe disturbance of consciousness in patients with non-traumatic cerebral hemorrhage (**A**) and cerebral infarction (**B**); (**C** and **D**) ROC curve analysis of TyG index predicting in-hospital mortality in patients with non-traumatic cerebral hemorrhage (**C**) and cerebral infarction (**D**)

Furthermore, we calculated the area under the ROC curve (AUC) to examine the TyG index’s ability to predict the severity of patients’ disturbance of consciousness and in-hospital mortality. The results showed that in patients with non-traumatic cerebral hemorrhage, the AUC of the TyG index to predict the severity of impaired consciousness was higher than 0.6 (IQR: 0.627 [0.551, 0.704]; numeric: 0.610 [0.528, 0.692]) (Fig. 4A), and the AUC predicting in-hospital mortality was higher than 0.65 (IQR: 0.664 [0.606, 0,722]; numeric: 0.661 [0.600, 0.721]) (Fig. 4C). The TyG index’s AUC for predicting the severity of consciousness disturbances in patients with cerebral infarction was higher than 0.55. (IQR: 0.579 [0.532, 0,626]; numeric: 0.571 [0.522, 0.620]) The AUC for predicting in-hospital mortality was greater than 0.6 (Fig. 4B) (IQR: 0.608 [0.556. 0.660]; numeric: 0.610 [0.556, 0.663]) (See **Fig. **4D). Altogether, The TyG index offers some predictive value for the severity of consciousness disturbances as well as in-hospital mortality in patients with the cerebrovascular disease.

**Fig. 5:**
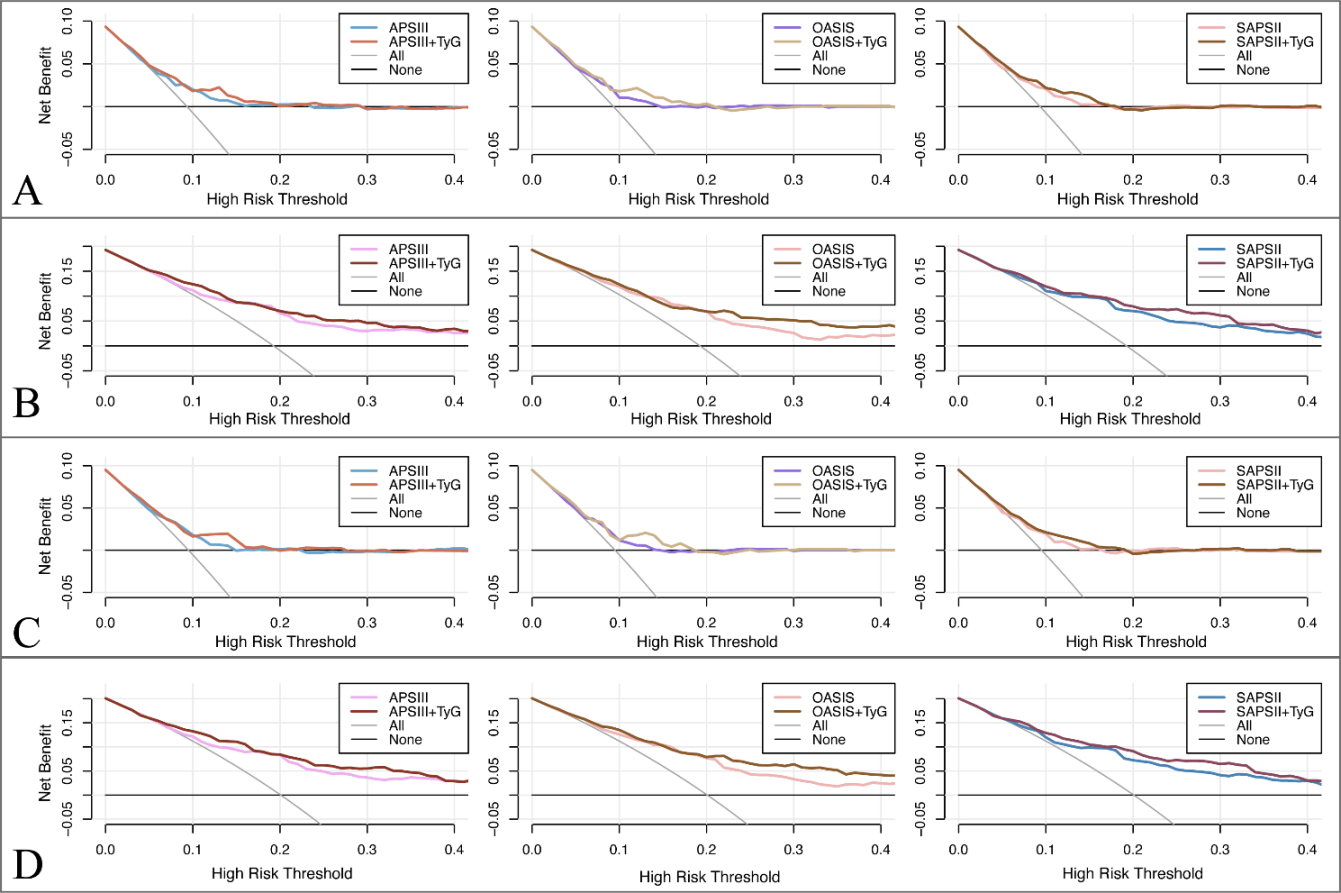
Decision curve analysis of scoring tools with and without considering the TyG index (IQR). (**A**) Predicting severe disturbance of consciousness in patients with non-traumatic cerebral hemorrhage. (**B**) Predicting in-hospital mortality in patients with non-traumatic cerebral hemorrhage. (**C**) Predicting severe disturbance of consciousness in patients with cerebral infarction. (**D**) Predicting in-hospital mortality in patients with cerebral infarction

In addition, we calculate the IDI of the scoring tools (APSIII, OASIS, SAPSII) when the TyG index is considered to analyze the impact of the TyG index on the predictive ability to score tools. IDI is a tool for assessment that responds to the degree of improvement in the predictive ability of the model, with a value greater than 0 indicating a positive improvement and a value less than 0 indicating a negative improvement. The results demonstrate that the predictive ability of the scoring tools with the TyG index has improved in comparison to those without the TyG index. After considering the TyG index according to quartile classification (TyG (IQR)), the predictive ability of the scoring tool for severe disturbance of consciousness and mortality was significantly improved (P < 0.05) in both non-traumatic cerebral haemorrhage and cerebral infarction patients. The predictive ability of the scoring tool for mortality was improved significantly (P < 0.05) when the numerical TyG index (TyG (numeric)) was considered for both, whereas the improvement in predictive ability for severe consciousness impairment was not statistically significant (P > 0.05) (Table 4). Moreover, we drew clinical decision curves to assess the clinical utility improvement of the TyG index. The results showed that the net clinical benefit of each scoring tool also had an improvement after considering the TyG index (Fig. 5).

**Table 4:** The incremental effect of the TyG index

Score	AUC [95% CI]	IDI [95% CI] (+ TyG (IQR))	P-value	IDI [95% CI] (+ TyG (numeric))	P-value
Severity of consciousness disturbance (patients with non-traumatic cerebral hemorrhage)					
APSIII	0.672 [0.594, 0.750]	0.0148 [0.0052, 0.0244]	**0.0025**	0.0016 [-0.0018, 0.0050]	0.3554
OASIS	0.641 [0.562, 0.719]	0.0162 [0.0054, 0.0271]	**0.0034**	0.0028 [-0.0017, 0.0073]	0.2262
SAPSII	0.654 [0.574, 0.733]	0.0156 [0.0048, 0.0264]	**0.0047**	0.0023 [-0.0021, 0.0067]	0.3099
In-hospital mortality (patients with non-traumatic cerebral hemorrhage)					
APSIII	0.736 [0.680, 0.793]	0.0385 [0.0188, 0.0581]	**0.0001**	0.0303 [0.0104, 0.0502]	**0.0029**
OASIS	0.746 [0.695, 0.797]	0.0560 [0.0329, 0.0791]	**< 0.0001**	0.0415 [0.0191, 0.0640]	**0.0003**
SAPSII	0.757 [0.704, 0.810]	0.0520 [0.0298, 0.0741]	**< 0.0001**	0.0449 [0.0217, 0.0681]	**0.0002**
Severity of consciousness disturbance (patients with cerebral infarction)					
APSIII	0.664 [0.584, 0.744]	0.0164 [0.0060, 0.0267]	**0.0020**	0.0018 [-0.0017, 0.0053]	0.3166
OASIS	0.625 [0.542, 0.708]	0.0176 [0.0057, 0.0294]	**0.0037**	0.0027 [-0.0019, 0.0074]	0.2452
SAPSII	0.643 [0.560, 0.726]	0.0164 [0.0044, 0.0283]	**0.0072**	0.0021 [-0.0023, 0.0064]	0.3508
In-hospital mortality (patients with cerebral infarction)					
APSIII	0.741 [0.683, 0.799]	0.0414 [0.0202, 0.0626]	**0.0001**	0.0367 [0.0138, 0.0597]	**0.0017**
OASIS	0.753 [0.700, 0.806]	0.0598 [0.0349, 0.0847]	**< 0.0001**	0.0489 [0.0236, 0.0742]	**0.0002**
SAPSII	0.750 [0.694, 0.806]	0.0567 [0.0325, 0.0809]	**< 0.0001**	0.0508 [0.0251, 0.0765]	**0.0001**

## Discussion

This study reveals for the first time that the TyG index can predict severe disturbance of consciousness in patients with critical cerebrovascular disease, and we also investigate the impact of the TyG index on the prognosis of these patients. The study demonstrates that in critically sick patients with cerebrovascular illness, high levels of the TyG index are a significant independent risk factor for the severity of consciousness disturbance and in-hospital mortality. Even after potential confounders were considered, this association still held true. Our findings also indicated that the degree of patients’ consciousness disturbance and in-hospital mortality were linearly related to the TyG index.

Cerebrovascular disease remains the main cause of mortality and disability worldwide, despite notable improvements in early diagnosis and treatment [19]. Type 2 diabetes is an established risk factor for cerebrovascular disease [20, 21]and is associated with the high risk of both ischemic and hemorrhagic strokes [22]. In type 2 diabetic patients, hyperglycemia is also linked to an increased risk of cerebrovascular diseases [22]. Currently available anti-diabetic drugs mainly play a role through two main mechanisms: insulin resistance (IR) and pancreatic β-cell dysfunction [23]. According to the evidence, IR, beta-cell dysfunction, and hyperglycemia affect the risk of cardiovascular disease independently of each other [24]. IR is not only a risk factor for the development of cardiovascular disease but is also associated with the prognosis of cardiovascular disease [25]. Whether or not they result in clinically significant vascular events, cardiovascular risk factors are linked to pathological brain damage associated with cognitive dysfunction [26, 27]. In addition, several pathophysiological processes suggest that hyperviscosity and increased nitrogenous waste due to metabolic disturbances may be potential mechanisms for IR to exacerbate disturbance consciousness in cerebrovascular disease patients. Nevertheless, there are no studies to verify this [13–17]. The TyG index is considered a credible and straightforward surrogate marker of IR for clinical practice [28]. A study of 6,091 patients showed that the TyG index had a higher predictive ability for metabolic syndrome than HOMA-IR (homeostatic model assessment to determine insulin resistance) (0.837 vs. 0.680, P-value < 0.001) [29]. In parallel, the fasting triglyceride and glucose data required to calculate the TyG index are easily accessible clinically, making the application of the TyG index simpler as well. However, the TyG index is affected by variables such as race and alcohol consumption. We therefore accounted for these confounding variables in the model’s adjustment.

The results of this study showed that the TyG index was predictive of the severity of consciousness disturbance and prognosis in both non-traumatic cerebral hemorrhage and cerebral infarction patients. There are currently very few studies on the impact of the TyG index on cerebrovascular patients’ in-hospital mortality and consciousness disturbance. An earlier long-term follow-up investigation revealed that a high TyG index level was a strong independent predictor of cerebrovascular events [30]. Several potential mechanisms have been suggested to explain the correlation between the TyG index and the development of cerebrovascular diseases. First, IR activates inflammation-related genes and interferes with insulin signaling [31, 32], leading to varying degrees of chronic inflammation, oxidative responses, and endothelial cell dysfunction to damage blood vessels [33, 34], leading to cerebrovascular disease. Secondly, in the IR state, the production of endothelial nitric oxide and the release of procoagulant factors lead to platelet aggregation and induce a prothrombotic state [35], which leads to disorders in the coagulation of the body and triggers cerebrovascular lesions accordingly [36, 37]. Lastly, IR induces prolonged endoplasmic reticulum stress and macrophage apoptosis, increases late vulnerable plaque formation, and causes plaque necrosis at the onset of atherosclerosis [32, 38]. Moreover, IR exacerbates the effects of other vascular risk factors, and leads to the development of cerebrovascular disease [9]. The progression of cerebrovascular disease then directly affects the severity of the patient’s consciousness disturbance and prognosis.

In summary, this study revealed the significant effect of the TyG index on the severity of consciousness disturbance and prognosis of patients with cerebrovascular disease. However, there are some other limitations of the present study that should be mentioned. Firstly, the results were not representative because all enrolled patients were from an American population. Secondly, due to the limitations of public databases, some potential confounders we did not include in the study such as dietary patterns, physical activity, long-term antihypertensive drugs use. Additionally, patients’ personal histories such as tobacco use, alcohol abuse, and long-term anticoagulant use were identified by ICD in this study. However, some patients with these personal histories were not documented by the clinician in the discharge or admission diagnosis. This may prevent us from identifying certain patients with tobacco use, alcohol abuse, or long-term anticoagulant use. Finally, due to the small sample size, the results from this study need to be validated in a larger cohort.

## Conclusion

Our study showed that the TyG index was a significant predictor of severe disturbance of consciousness and prognosis in critically ill patients with cerebrovascular disease, associated with the risk of severe disorder of consciousness and in-hospital mortality in an approximately linear relationship. Moreover, the TyG index offers some predictive value for severe consciousness disturbances as well as in-hospital mortality in patients with cerebrovascular disease, which may increase the accuracy of identifying high-risk patients.

## List of abbreviations

MIMIC-IV: Medical Information Mart for Intensive Care IV
TyG index: Triglyceride-glucose index
ICU: Intensive care unit
ROC: Receiver operating characteristic
IR: Insulin resistance
ICD-10: International Classification of Diseases, Tenth Revision
ICD-9: Based on the International Classification of Diseases, Ninth Revision
GCS: Glasgow coma scale
SQL: Structured query language
BMI: Body mass index
CPK: Creatine phosphokinase
CKMB: Creatine kinase isoenzyme MB
IDH: Lactate dehydrogenase
ALP: Alkaline phosphatase
AST: Aspartate aminotransferase
INR: International normalized ratio
ALT: Alanine aminotransferase
PT: Prothrombin Time
PTT: Active partial thromboplastin time
CKD: Chronic kidney disease
SD: Standard deviation
IQR: Interquartile range
ORs: Odds ratios
CI: Confidence intervals
HbA 1c: Glycated hemoglobin A 1c
APSIII: Acute physiology score III
OASIS: Oxford acute severity of illness score
SAPSII: Simplified acute physiology score
